# Novel Drug Screening Assay for *Acanthamoeba castellanii* and the Anti-Amoebic Effect of Carbonic Anhydrase Inhibitors

**DOI:** 10.1021/acs.jmedchem.3c01020

**Published:** 2023-12-27

**Authors:** Susanna Haapanen, Harlan Barker, Fabrizio Carta, Claudiu T. Supuran, Seppo Parkkila

**Affiliations:** † Faculty of Medicine and Health Technology, Tampere University, FI-33520 Tampere, Finland; ‡ Fimlab Ltd, Tampere University Hospital, FI-33520 Tampere, Finland; § Neurofarba Department, Sezione di Chimica Farmaceutica e Nutraceutica, Università degli Studi di Firenze, Via U. Schiff 6, Sesto Fiorentino, I-50019 Firenze, Italy

## Abstract

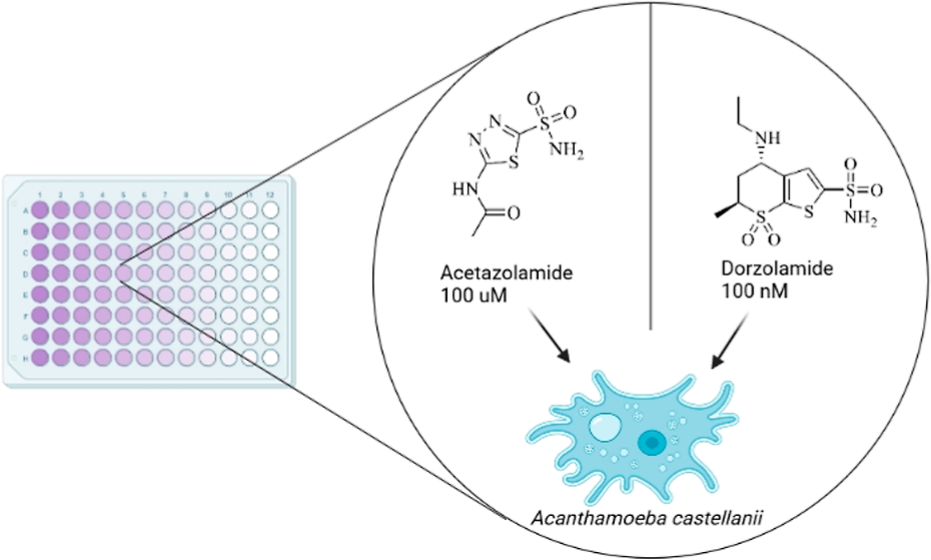

*Acanthamoeba castellanii* is an amoeba that inhabits soil and water in every part of the world. Acanthamoeba infection of the eye causes keratitis and can lead to a loss of vision. Current treatment options are only moderately effective, have multiple harmful side effects, and are tedious. In our study, we developed a novel drug screening method to define the inhibitory properties of potential new drugs against *A. castellanii* in vitro. We found that the clinically used carbonic anhydrase inhibitors, acetazolamide, ethoxzolamide, and dorzolamide, have promising antiamoebic properties.

## Introduction

**Figure 1:**
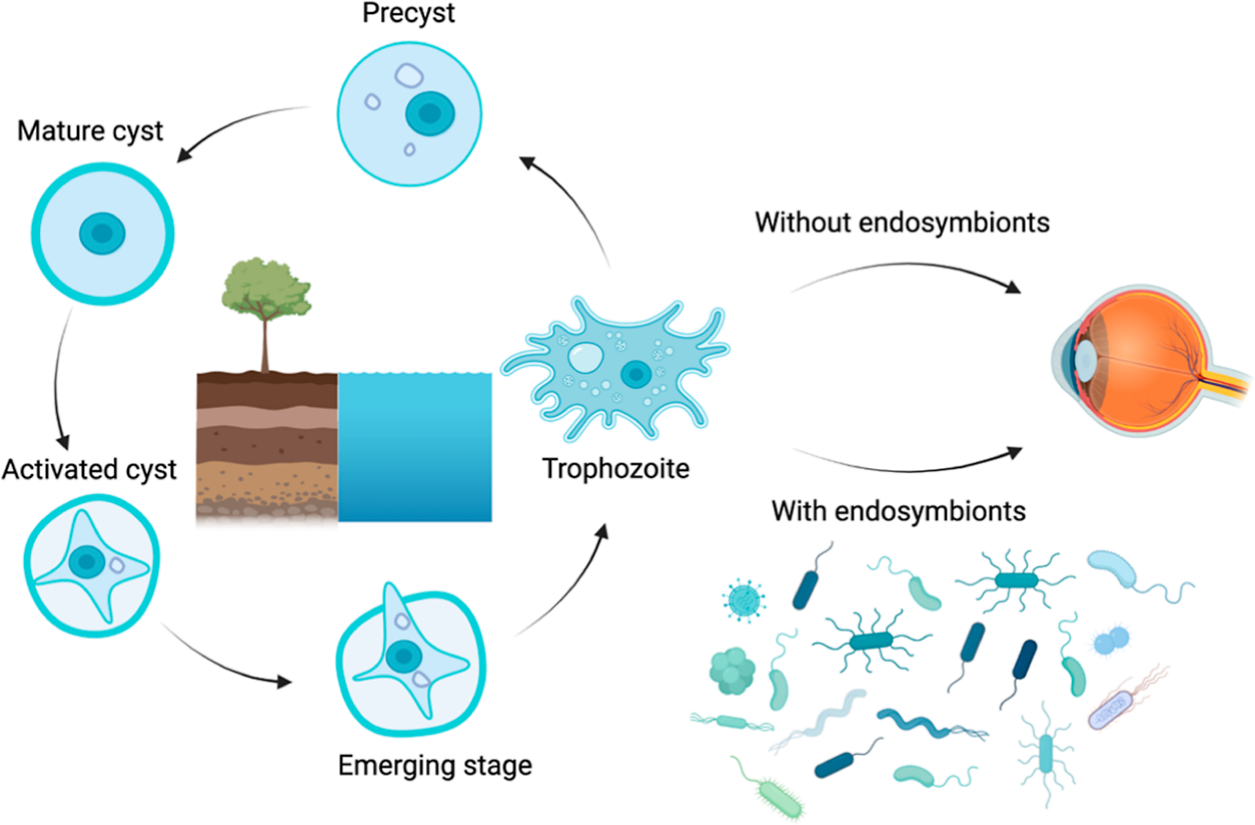
Life cycle of *Acanthamoeba castellanii*. *A. castellanii* lives in water and soil and often harbors endosymbionts.

*Acanthamoeba castellanii* is an opportunistic amoeba ubiquitously present in soil and natural water.^1,2^ In humans, it causes sight-threatening keratitis named acanthamoeba keratitis (AK),^3,4^ and in immunocompromised patients, it seldom causes severe invasive infections such as granulomatous amoebic encephalitis (GAE).^5−7^ AK usually is contracted by individuals particularly exposed to risk factors, such as contact lens wearers,^8,9^ glucocorticoid eye drops users^10^ and patients recovering from eye operations, with the first group being the largest as accounts for over 150 million contact lens users worldwide.^11^ The annual incidence of AK is approximately 1.2 million in Western countries alone, causing loss of quality-adjusted life years due to complications subsequent to infection, e.g., monocular blindness.^12,13^*A. castellanii* has two distinct life cycle stages: (i) the metabolically active trophozoite and (ii) the cyst, which is a resistant and quiescent form of the parasite^14^ (Figure 1).

The cysts of *A. castellanii* are highly resistant to clinically used antimicrobial agents and to contact lens disinfectants^15,16^ mainly due to the protective effect of a double wall structure made of cellulose and other complex polysaccharides from adverse environmental factors.^17^ The cysts can live up to 25 years while retaining infectious capacity^16^ as they are capable of excystation once adverse environmental agents have receded, and that represents a main point of failure in the pharmacological treatment of AK.^12^ In addition, the trophozoites can form a biofilm on the surface of the contact lens, thus creating a protective layer from disinfectants.^18^

A comprehensive treatment against AK has not been established.^19^ Most medications are topically administered in the form of eye drops applied every hour for the first few days and then hourly during waking hours for several weeks.^12^ They include biguanide derivatives (e.g., polyhexamethylene biguanide hydrochloride and chlorhexidine gluconate^20^), and diamidine derivatives (e.g., propamidine and hexamidine isethionate^15,19^). They are either used as monotherapy or in combination with antibacterial or antifungal agents.^21^ Later, if the infection improves, the administration frequency can be reduced to once in 3 h.^15^ Overall AK directed pharmacological treatment may last 3–4 weeks, although it can last as long as 12 months.^12^ The restrictive and time-consuming features of the treatment may result in low compliance from patients. Besides the damage caused to the corneal tissue by *A. castellanii*,^12^ the risk for adverse side effects is present, such as loss of corneal tissue regeneration, corneal ulceration, scleritis, iris atrophy, and glaucoma,^15^ and becomes higher as the treatment course is extended. Additionally, there is evidence in clinical samples of *A. castellanii* strains that show significant drug resistance against conventional medication, e.g., polyhexamethylene biguanide hydrochloride.^22^ New treatment options are being developed that, thus far, show a moderate effect against cysts in vitro studies and among others include several quinazolinones,^23^ cobalt nanoparticles,^24^ and silver nanoparticles.^25^ However, these compounds have not been clinically tested^23−25^ and thus their efficacy in humans is yet to be discovered.

In this study, we focused our attention on carbonic anhydrases (CAs; EC 4.2.1.1) expressed in *A. castellanii*. We consider them as potential new targets in the fight against these pathogenic organisms.

Eight CAs from three different families are present in the genome of *A. castellanii*: three α-, three β- and two γ-CAs (Table 1).^26,27^ Both γ-CAs have been identified to be part of mitochondrial complex 1, suggesting they have an essential role in the utilization of energy in the cell.^28,29^ β-CAs are predicted to exist as mitochondrial, cytoplasmic, and transmembrane isoforms, suggesting a contribution to various actions in cell metabolism. All of the α-CAs are probably membrane-associated. Only α-CAs are found in the human genome, thus opening an exciting opportunity for specifically targeting the β- and γ-CAs of *A. castellanii* with inhibitors specific to these enzyme families.

**Table 1:** CAs of *A. castellanii*

CA (gene name)	entry ID	amino acid count	subcellular location^a^
α-CA (ACA1_060250)	L8GXK3	348	transmembrane
α-CA (ACA1_130470)	L8GPJ9	314	transmembrane
α-CA (ACA1_185050)	L8H518	279	transmembrane
β-CA (ACA1_164750)	L8GR38	288	mitochondrial
β-CA (ACA1_278940)	L8H861	280	transmembrane
β-CA (ACA1_365670)	L8GLS7	244	cytoplasmic
γ-CA (ACA1_260080)	L8GFM8	282	mitochondrial
γ-CA (ACA1_296480)	L8HK20	272	mitochondrial

a The sequences were retrieved from UniProt (entry IDs), and for six CAs the subcellular locations were predicted using DeepTMHMM-tool (https://www.biorxiv.org/content/10.1101/2022.04.08.487609v1.abstract) and TargetP 2.0 (https://services.healthtech.dtu.dk/service.php?TargetP-2.0). Subcellular location for γ-CAs are according to Gawryluk, Gray^28^ and Gawryluk, Chisholm, Pinto, Gray.^29^

*A. castellanii* is known to harbor other microbes as endosymbionts, including bacteria, fungi, and viruses, of which many are pathogenic. Proteobacteria and Actinobacteria are the most abundant phyla of AK endosymbiont bacteria (Table 2), and Pandoraviridiae and Mimiviridae are the most plentiful among viruses.^30^ Many different *A. castellanii* endosymbionts are found in corneal scrapings from AK patients; however, even more, have been found in samples from other locations, such as water and soil.^31^ Importantly, a majority of isolated clinical *A. castellanii* samples have included one or more endosymbionts.^32^ For instance, *Pseudomonas aeruginosa* causes difficult-to-treat keratitis on its own and was shown by Gu et al. to be present in over 70% of investigated clinical AK samples in their study.^30^ This coinfection is believed to progress the destruction of the cornea at a rate greater than that of a single infection with either of the pathogens.^30^

**Table 2:** Recognized Bacterial and Fungal Endosymbionts in AK According to Horn, Wagner,^37^ Barker, Brown,^38^ and Rayamajhee et al.^31^^a^

organism	phylum	detected in AK	strains count	all CA count	unique CA count
<>Achromobacter<> sp	Proteobacteria	yes^c^	20	40	7
<>Agrobacterium tumefaciens<>	Proteobacteria		6	19	3
<>Afipia felis<>	Proteobacteria		1	4	3
<>Brevibacillus<> sp	Firmicutes	yes^c^	24	25	6
<>Brevundimonas vesicularis<>	Proteobacteria	yes^c^	1	6	3
<>Burkholderia cepacia<>	Proteobacteria		4	31	6
<>Burkholderia pickettii<>	Proteobacteria		4	21	5
<>Burkholderia pseudomallei<>	Proteobacteria		15	39	3
<>Caedibacter<>	Proteobacteria		3	4	4
<>Amoebophilus asiaticus<>	Bacteroidetes	yes^g^	0	0	0
*Candidatus Babela massiliensis*	*Candidatus* Dependentiae	yes^h^	0	0	0
*Candidatus Caedibacter acanthamoebae*	Proteobacteria		1	1	1
<>Candidatus Odyssella<>	Proteobacteria		1	3	3
<>Candidatus Paracaedibacter<> spp	Proteobacteria	yes^b^^,^^e^	1	1	1
<>Candidatus Procabacter<>	Proteobacteria		0	0	0
*Candidatus Protochlamydia amoebophila*	Chlamydiae		1	2	2
<>Chlamydia trachomatis<>	Chlamydiae	yes^d^^,^^f^	0	0	0
<>Chlamydophila pneumoniae<>	Chlamydiae		0	0	0
<>Coxiella burnetii<>	Proteobacteria		3	4	2
<>Cytophaga<> spp	Bacteroidetes		4	11	6
<>Escherichia coli<>	Proteobacteria	yes^c^	4	35	4
<>Francisella tularensis<>	Proteobacteria		6	15	2
<>Helicobacter pylori<>	Campylobacterota		3	48	2
<>Legionella cherrii<>	Proteobacteria	yes^f^	1	3	3
<>Legionella dumoffii<>	Proteobacteria	yes^f^	0	0	0
<>Legionella lytica<>	Proteobacteria	yes^f^	0	0	0
<>Legionella pneumophila<>	Proteobacteria	^yes^^d^^,^^f^	4	27	5
<>Listeria monocytogenes<>	Firmicutes		8	23	2
<>Methylophilus<> spp	Proteobacteria		5	12	5
<>Microbacterium<> sp	Actinobacteria	^yes^^c^	35	50	26
<>Mobiluncus curtisii<>	Actinobacteria		3	3	1
<>Mycobacterium avium<>	Actinobacteria	^yes^^d^^,^^f^	16	23	5
<>Mycobacterium bovis<>	Actinobacteria	^yes^^d^^,^^f^	2	7	3
<>Mycobacterium tuberculosis<>	Actinobacteria	^yes^^d^^,^^f^	20	47	5
<>Neochlamydia<>	Chlamydiae		3	3	1
<>Parachlamydia acanthamoebae<>	Chlamydiae	yes^e^	2	2	1
<>Pseudomonas aeruginosa<>	Proteobacteria	yes^c^^,^^d^^,^^f^	3	29	3
<>Stenotrophomonas geniculata<>	Proteobacteria	yes^f^	1	2	2
<>Rickettsia<>	Proteobacteria	yes^d^	23	25	4
<>Salmonella enterica<>	Proteobacteria		31	33	2
<>Simkania negevensis<>	Chlamydiae		1	1	1
<>Stenotrophomonas maltophilia<>	Proteobacteria	yes^c^	4	25	1
<>Vibrio cholerae<>	Proteobacteria		9	29	2
<>Waddlia chondrophila<>	Chlamydiae		2	2	1
<>Blastomyces dermatitidis<>	Ascomycota		4	11	3
<>Cryptococcus neoformans<>	Basidiomycota	yes^d^	6	12	2
<>Histoplasma capsulatum<>	Ascomycota		5	25	7
<>Sporothrix schenckii<>	Ascomycota		2	12	6

a “Strains count”—number of unique strains or subspecies found for the target organism. “All CA count”—number of unique CA proteins for that organism (possessing a UniPROT protein ID). “Unique CA count”—from the “All CA count” proteins, how many are left after reducing to a max 80% similarity.

b Refrence (39).

c Reference (40).

d Reference (30).

e Reference (41).

f Reference (32).

g Reference (42).

h Reference (43).

Most of the bacterial endosymbionts are believed to increase the pathogenicity of *A. castellanii* as different endosymbiont-containing strains had a significantly greater cytopathic effect on fibroblast monolayers than noninfected amoebae.^33^ In addition, endosymbionts are suspected to increase the virulence of *A. castellanii*, possibly through horizontal gene transfer.^30,34^ Contradicting insights have been presented, however, and the pathogenicity of the endosymbiont might affect whether the virulence of *A. castellanii* increases.^35^

## Results

**Figure 2:**
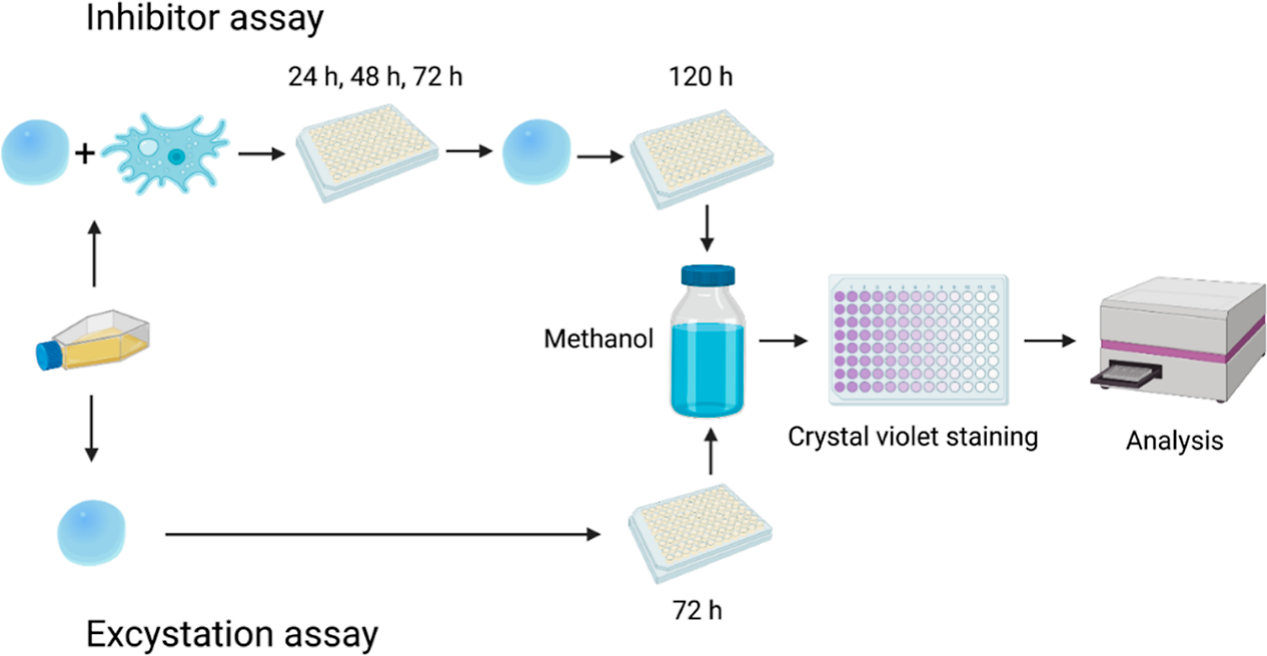
Protocol of the inhibitor assay and excystation assay of the drug screening method. In the inhibitor assay, both trophozoites and cysts are investigated, while in an excystation assay, only cysts are exposed to the inhibitor effect.

**Figure 3:**
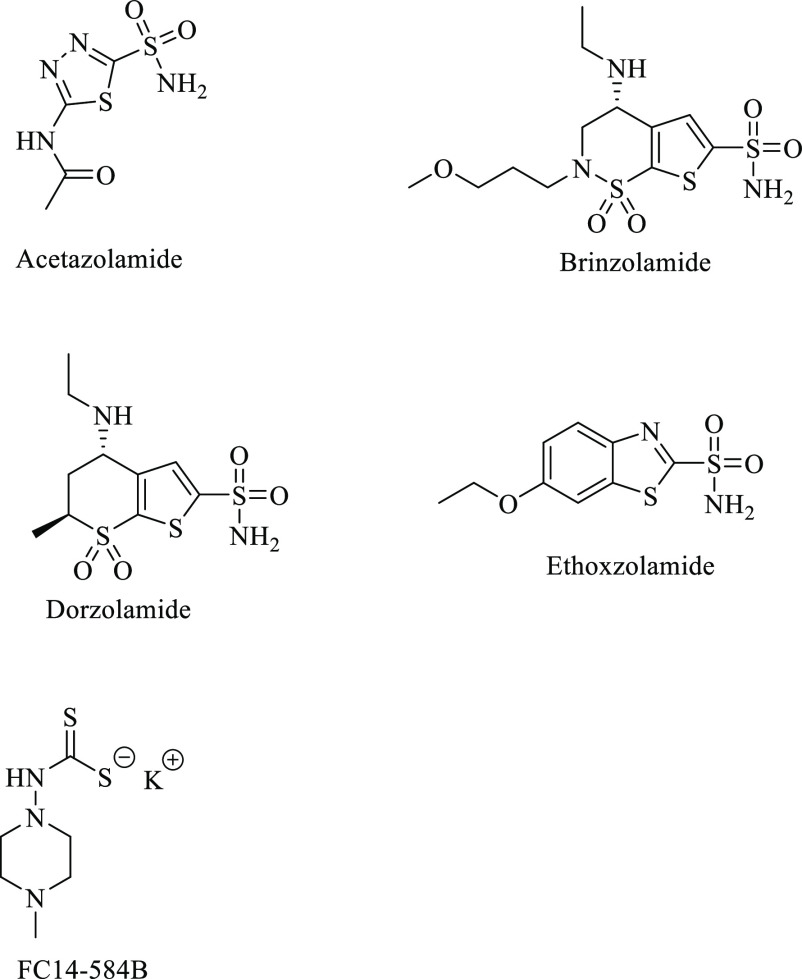
Chemical structures of the tested inhibitors. Acetazolamide, brinzolamide, dorzolamide, and ethoxzolamide are sulfonamides; FC14-584B is a dithiocarbamate.

We designed a novel drug screening method to define the inhibitory properties of potential new drugs against *A. castellanii* in vitro (Figures 2 and 3). The method consists of two independent assays: an inhibitor assay and an excystation assay. Using the inhibitor assay, we tested the inhibitors against trophozoites and the ability of cysts to remain viable after the inhibitor effect. With the excystation assay, we tested the ability of cysts to perform excystation in the presence of an inhibitor to investigate how the selected drugs may affect the transformation of cysts to active trophozoites. Using this new method, we aimed to find potential CAIs to treat AK and invasive infections caused by *A. castellanii*.

**Figure 4:**
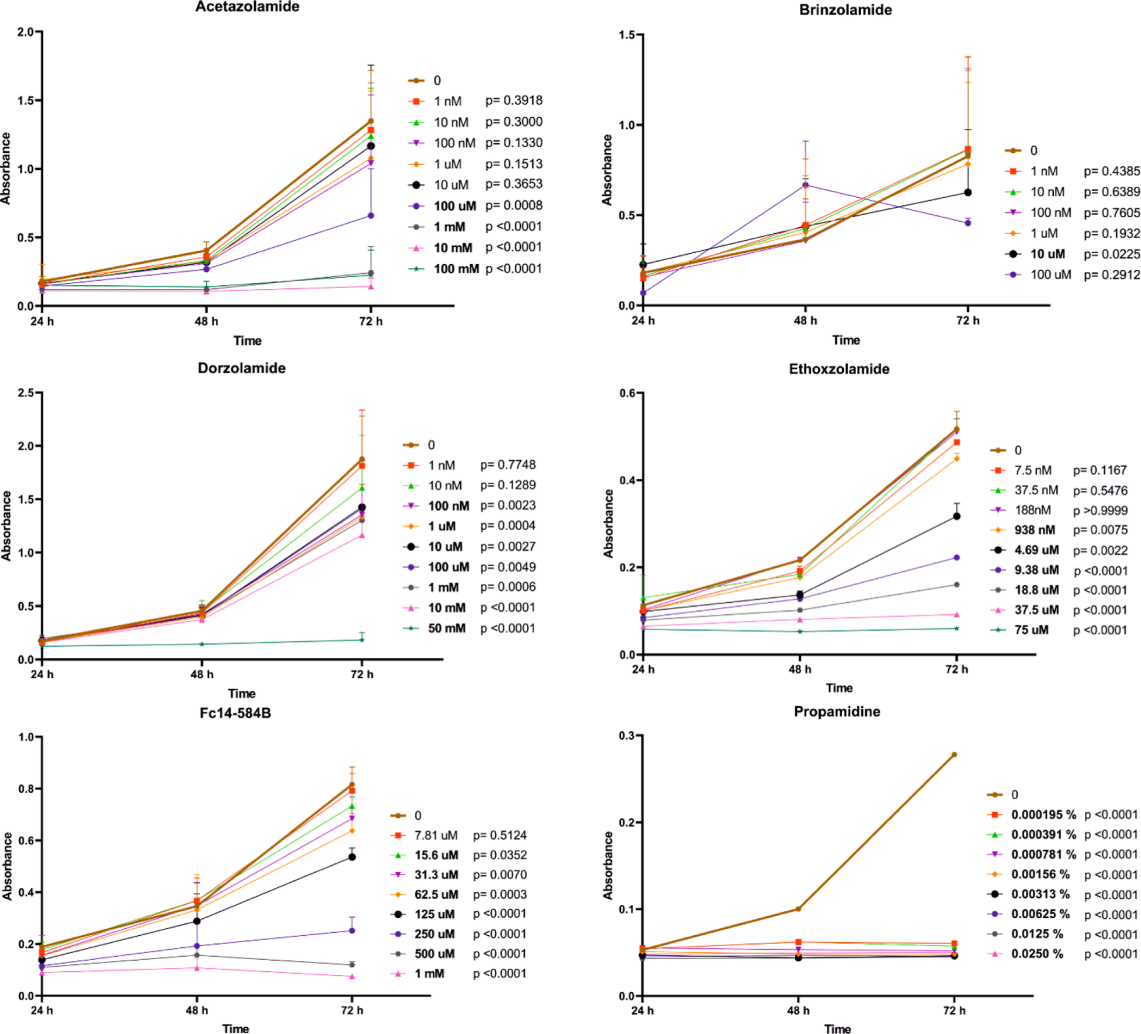
Determination of trophozoites growth inhibition with various concentrations of six different compounds in *A. castellanii* culture. Cell density was determined by optical scanning of crystal violet staining (absorbance) at three-time points (24, 48, and 72 h). Unpaired *t*-test was used to compare each concentration against the control (0), at the 72 h time point (bold: *p*-value < 0.05). All tested inhibitors, except brinzolamide, were discovered to decrease the viability of the trophozoites. Propamidine is presented as percentage values because the eye drop used is 0.1% concentration. The number of sample replicates were 11 for acetazolamide, 11 for brinzolamide, 15 for dorzolamide, 6 for ethoxzolamide, 7 for Fc14-584B and 6 for propamidine. Whiskers represent standard deviation (SD).

As a commonly used therapeutic agent, propamidine stands as a comparison agent in this study. Only brinzolamide showed no inhibitory effect on trophozoites or cysts. This is in contrast to the other inhibitors tested, which reduced the number of viable trophozoites (Figure 4). Fc14-584B and acetazolamide inhibited the growth of trophozoites at concentrations of 15.6 and 100 μM, respectively. Ethoxzolamide and dorzolamide were even more effective, as they restricted the growth of trophozoites at 938 and 100 nM concentrations, respectively.

**Figure 5:**
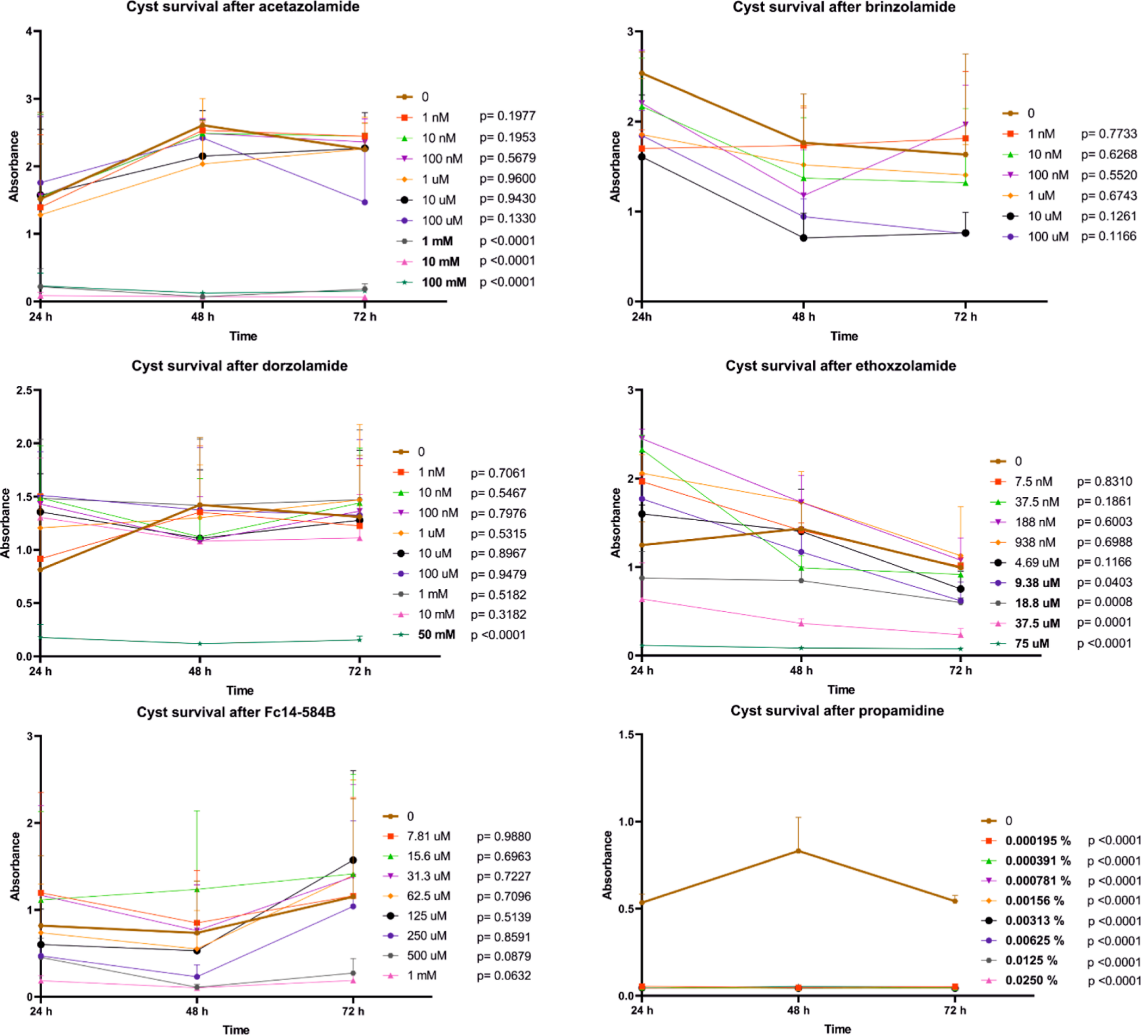
Cyst survival assay shows the ability of cysts to excystate after the effect of various concentrations of six different compounds in an *A. castellanii* culture. The cyst survival was measured indirectly as the staining can only detect trophozoites. Cell density was determined by optical scanning of crystal violet staining (absorbance) at three-time points (24, 48, and 72 h). Unpaired *t*-test was used to compare each concentration against the control (0), at the 72 h time point (bold: *p*-value < 0.05). All tested inhibitors, except brinzolamide and Fc4-584B, were discovered to decrease the ability of the cysts to transform into trophozoites. Propamidine is presented as percentage values because the eye drop used is 0.1% concentration. The number of sample replicates were 11 for acetazolamide, 11 for brinzolamide, 15 for dorzolamide, 6 for ethoxzolamide, 7 for Fc14-584B and 6 for propamidine. Whiskers represent standard deviation (SD).

Ethoxzolamide, acetazolamide, and dorzolamide had effects on cyst survival, but at higher concentrations than on survival of trophozoites; they were effective at 9.38 μM, 1 mM and 50 mM concentrations, respectively. Fc14-584B had no statistically significant effect on cyst survival, although the shape of growth curves points to a reduction of cyst survival at a concentration ≥500 μM (Figure 5).

**Figure 6:**
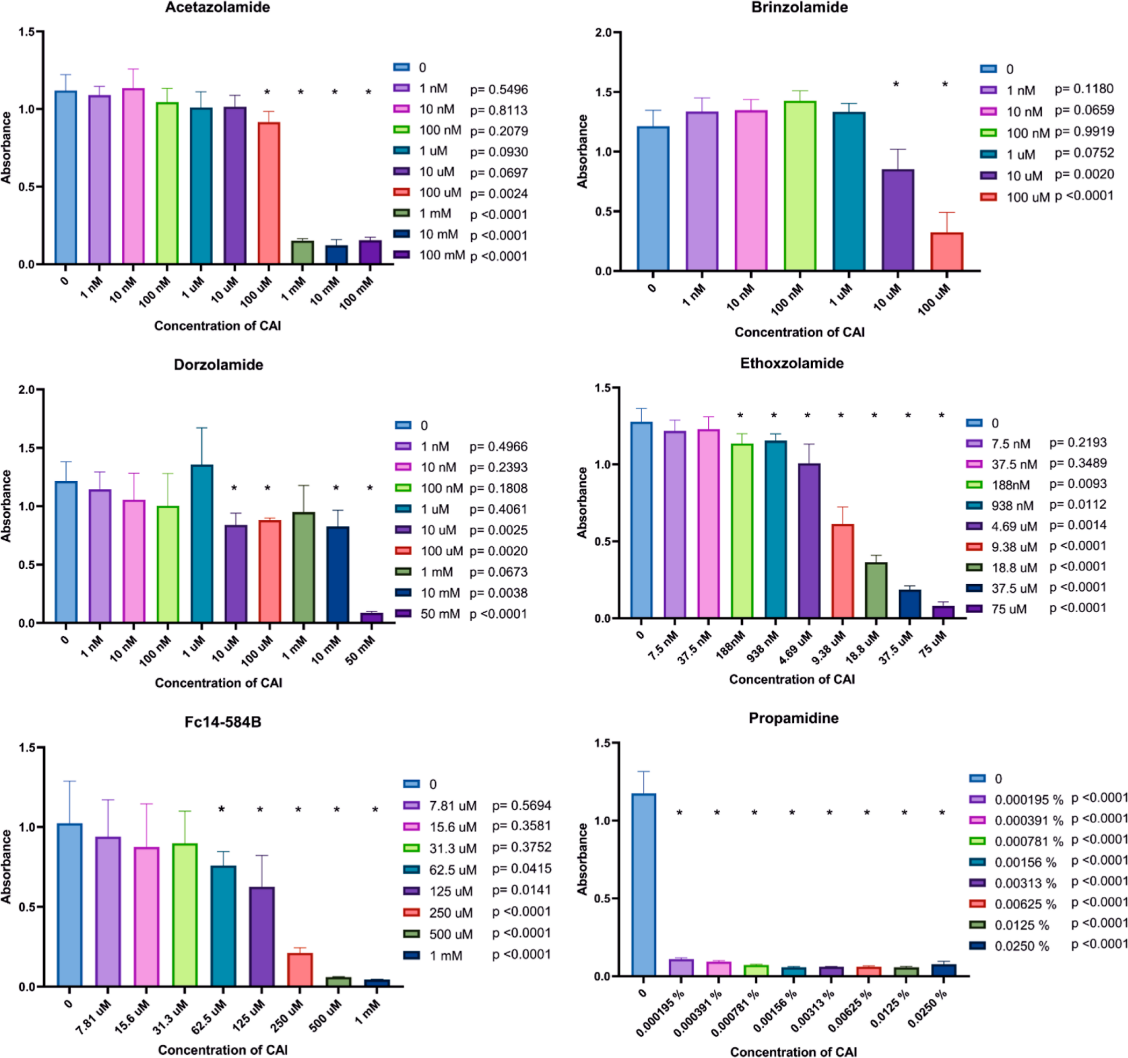
Excystation assay indirectly displays the ability of cysts to excystate and act as an active trophozoite with various concentrations of six different compounds in *A. castellanii* culture. Cell density was determined by optical scanning of crystal violet staining (absorbance) at one-time point (72 h). Unpaired *t*-test was used to compare each concentration against the control (0) (*: *p*-value < 0.05). All tested inhibitors were discovered to decrease the ability of the cysts to excystate. Propamidine is presented as percentage values because the eye drop used is 0.1% concentration in the presence of inhibitor. The number of sample replicates was 6 for each inhibitor. Whiskers represent standard deviation (SD).

The excystation assay results are roughly equivalent to the results of the inhibitor assay (Figure 6), except for brinzolamide and Fc14-584B being able to inhibit the excystation at high concentrations (brinzolamide ≥10 μM and Fc14-584B ≥ 62.5 μM). Ethoxzolamide, dorzolamide, and acetazolamide were effective against excystation at 188 nM, 10 μM, and 100 μM, respectively. Ethoxzolamide was found to be even more effective in inhibiting excystation than the growth of trophozoites.

We calculated interassay coefficients of variation for the negative controls of the assay. In the first part of inhibitor assay, the coefficients of variation are 19.4% for 24 h, 28.7% for 48 h, and 21.4% for 72 h for acetazolamide, 8.5, 11.2, and 11.8%, respectively, for dorzolamide, 8.8, 13.0, and 6.8%, respectively, for brinzolamide, 6.9, 13.9, and 7.0%, respectively, for Fc14-584B, 3.2, 4.1, and 4.1%, respectively, for ethoxzolamide, and 1.9, 1.0, and 7.9%, respectively, for propamidine. The coefficients of variation in cyst survival part are 10.2% for 24 h, 1.8% for 48 h, and 8.6% for 72 h for acetazolamide. The respective values for dorzolamide are 54.2, 42.6, and 33.3%, for brinzolamide 7.8, 12.6, and 14.6%, for Fc14-584B 44.2, 23.2, and 28.1%, for ethoxzolamide 12.3, 13.1, and 7.4%, and for propamidine 9.0, 23.1, and 6.0%. The coefficients of variation for excystation assay are 9.5% for acetazolamide, 8.6% for dorzolamide, 2.7% for brinzolamide, 15.5% for Fc14-584B, 2.8% for ethoxzolamide, and 7.2% for propamidine.

**Figure 7:**
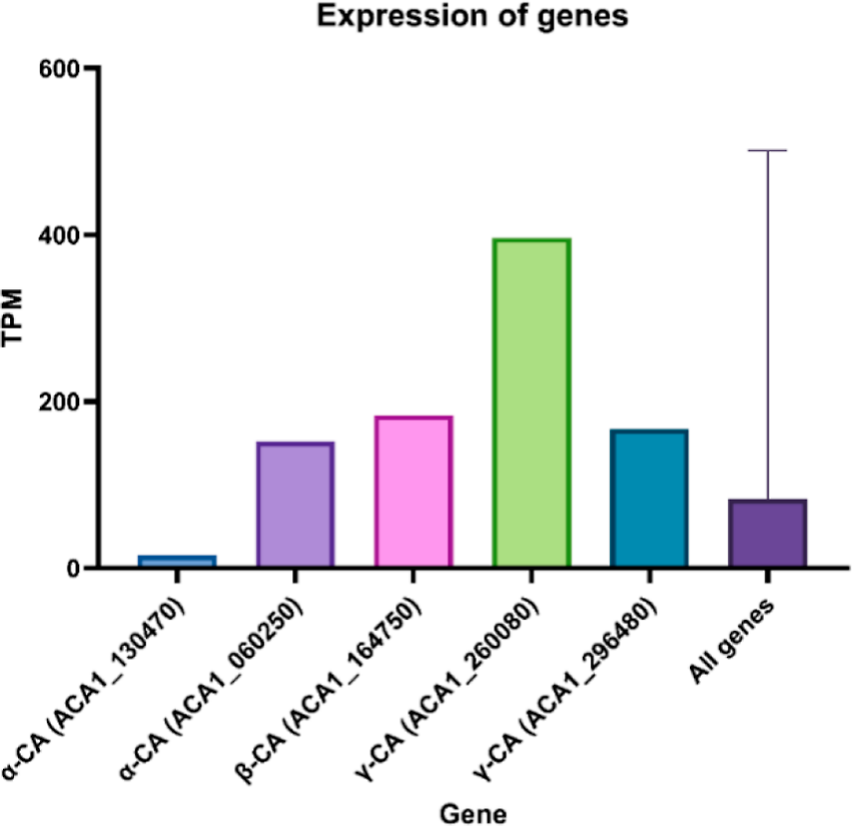
Basal expression levels of five of the eight known CAs of *A. castellanii*, based on RNA-Seq data (control sample, König et al.).^36^ Values are given as transcripts per million (TPM), converted from RPKM; UniProt gene identifiers are used.

To identify the key CAs endogenously expressed in *A. castellanii*, we analyzed mRNA sequence data from König et al.,^36^ describing the expression of five CAs of *A. castellanii* (Figure 7). This data shows that γ-CA (ACA1_260080) has the highest expression level (∼400 TPM), suggesting an essential role in cell metabolism. Conversely, α-CA (ACA1_130470) has a comparatively low expression level (∼15 TPM). Expression values of the other CAs are above the mean expressions of all genes (83.0 TPMs) and can be considered moderate.

We found that 22 bacteria and fungi were isolated from a clinical *A. castellanii* sample (Table 2). In addition, many environmental samples inhabited endosymbionts. Only a few endosymbionts have no CAs in their genome, in contrast to some of them having several dozen CAs.

**Figure 8:**
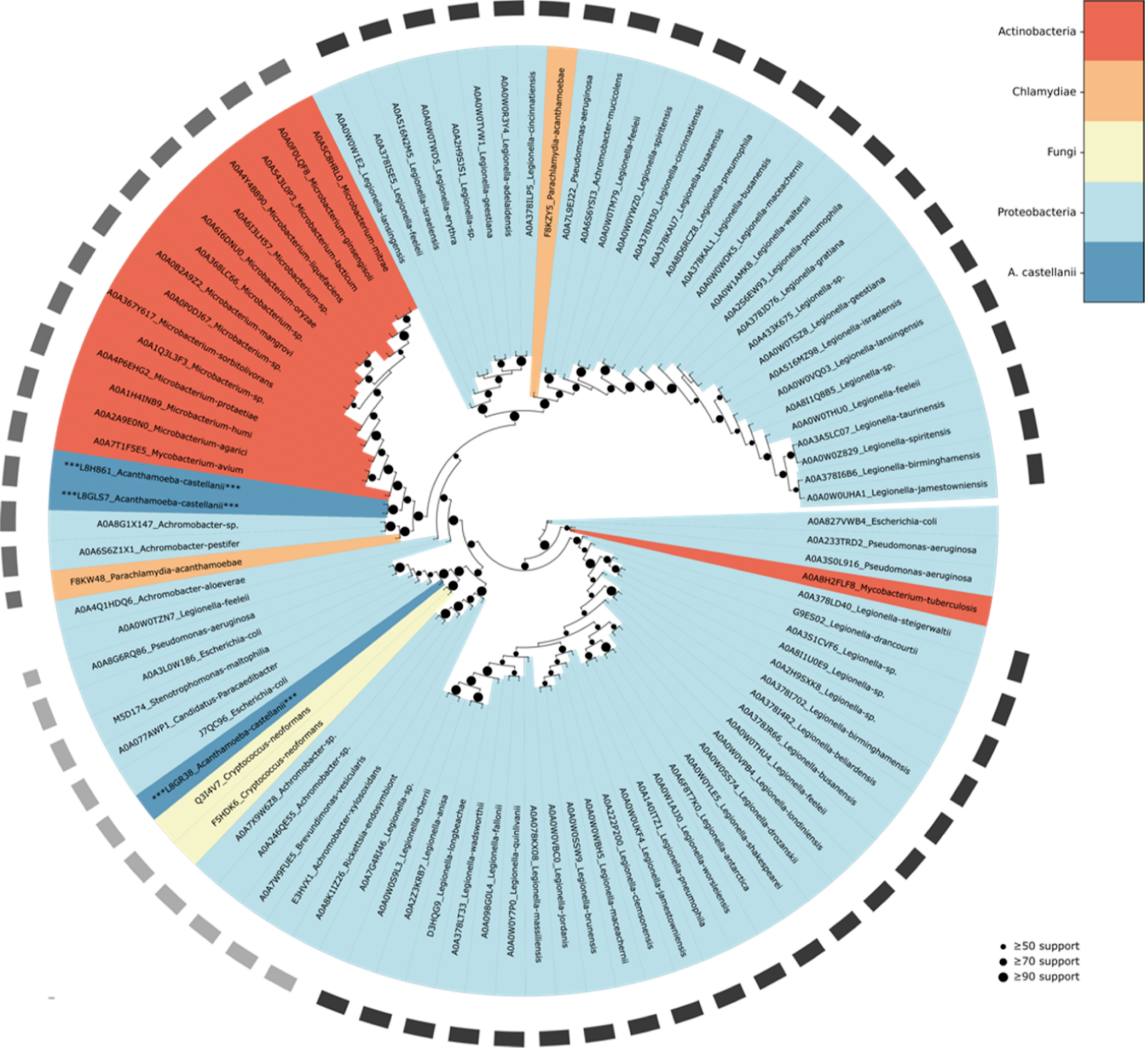
Phylogenetic inference shows the relatedness of *A. castellanii* and endosymbiont β-CAs. A total of 3 *A. castellanii* and 95 endosymbiont β-CA protein sequences were identified by BLAST search and subsequently analyzed for relatedness by maximum likelihood-based phylogenetic inference, with IQ-TREE.^44^ The resulting consensus tree was visualized with the ETE toolkit^45^ (vers. 3.1.2). Organism phyla are indicated by colors, as defined in the legend, and circle size at nodes represents the percentage of replicates supporting that branch topology.

**Figure 9:**
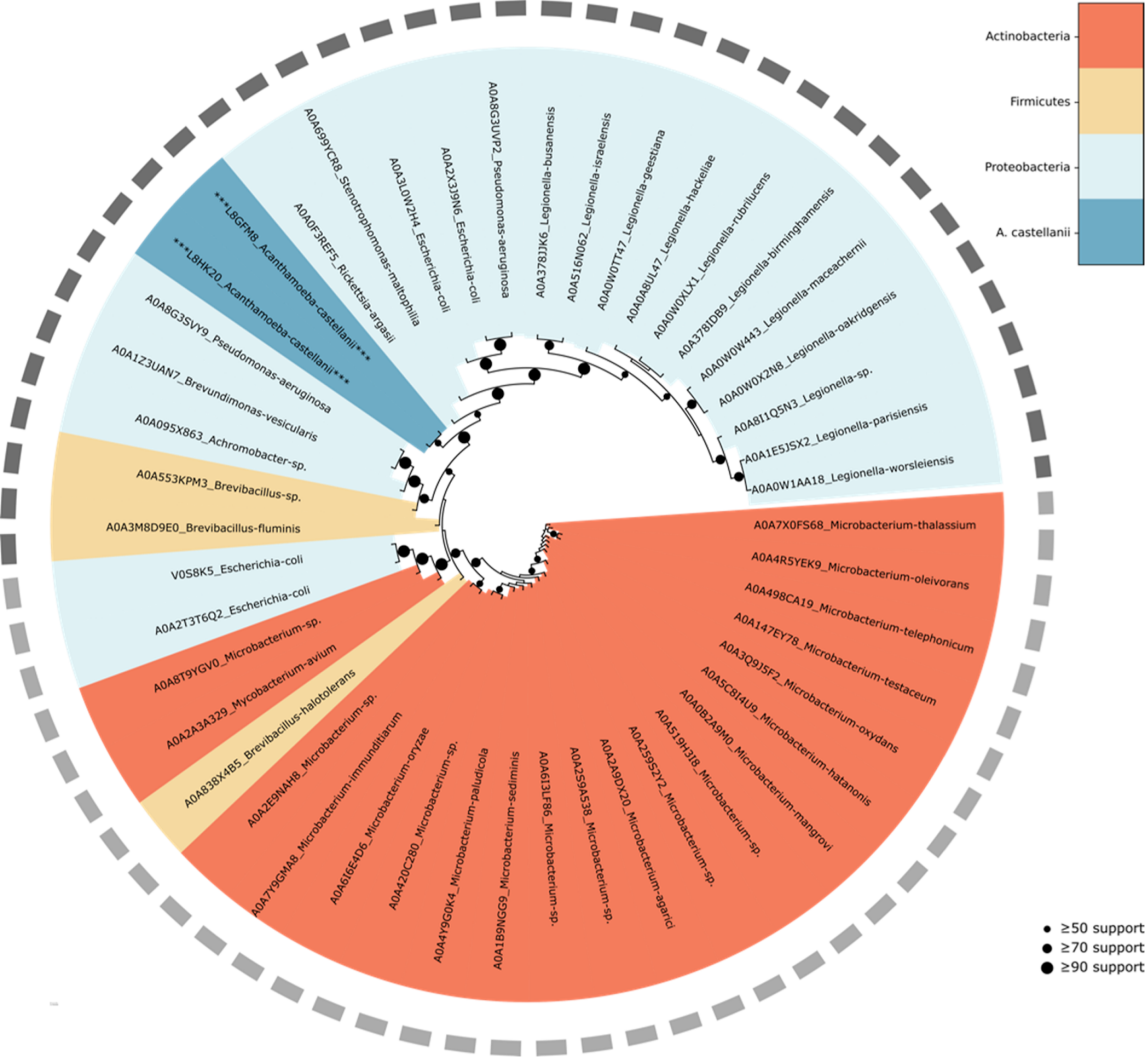
Phylogenetic inference shows the relatedness of *A. castellanii* and endosymbiont γ-CAs. A total of 2 *A. castellanii* and 46 endosymbiont γ-CA protein sequences were identified by BLAST search and subsequently analyzed for relatedness by maximum likelihood-based phylogenetic inference, with IQ-TREE.^44^ The resulting consensus tree subsequently visualized with the ETE toolkit^45^ (vers. 3.1.2). Organism phyla are indicated by colors, as defined in the legend, and circle size at nodes represent the percentage of replicates supporting that branch topology.

Maximum likelihood-based inference of phylogenetic relationships among 8 *A. castellanii* CAs combined with 38 endosymbiont CAs was performed using IQ-TREE. The resulting tree was visualized with the ETE toolkit and is presented as Figure 8. Likewise, phylogenetic relationships among two *A. castellanii* γ-CAs combined with 46 endosymbiont γ-CAs was performed and presented as Figure 9.

Within the β-CA tree, we observe that two of the *A. castellanii* CAs segregate together within a clade of β-CA proteins in actinobacteria organisms (most closely *Mycobacterium avium*), while the third *A. castellanii* CA co-occurs with proteobacteria β-CAs (most closely *Escherichia coli*). Within the γ-CA tree, the *A. castellanii* CAs again segregate together, and within a clade of proteobacteria (most closely with *Rickettsia argasii*).

## Discussion and Conclusions

Our new biphasic crystal violet staining-based method identified CAIs with the potential to treat infections of *A. castellanii*. Dorzolamide, acetazolamide, and ethoxzolamide all showed an excellent ability to interfere with the viability of both trophozoites and cysts. Dorzolamide and acetazolamide are especially compelling because of their longstanding clinical use in the treatment of glaucoma (both CAIs),^46,47^ epilepsy, and mountain sickness (acetazolamide).^48^

Dorzolamide is commercially distributed as an eye drop at a concentration of 20 mg/mL, equivalent to 55.4 mM. As a topical drug, the concentration in the eye is at a millimolar level at application. Our study used a 50 mM solution as the highest concentration to mimic the effect of the clinical product.

Acetazolamide can be administered orally, intravenously, or intramuscularly and in varying doses: usually between 250 to 1000 mg per day, regardless of the route of administration. Larsson and Alm have determined the concentrations of acetazolamide in blood with different oral doses: 31.3 mg correlates to 8.1 μM concentration in blood, 62.5 mg to 19.3 μM, and 250 mg to 72.0 μM.^49^ If it is assumed that the concentration in tear fluid is equivalent to the concentration in blood, we would nearly achieve a high enough concentration with a single oral dose of 250 mg as 100 μM is effective against *A. castellanii*. However, to our knowledge, there are no studies that measured the acetazolamide concentration equivalence between serum and aqueous humor. Conventionally, the suggested single dose of acetazolamide is 250–375 mg orally or intravenously. Intravenously, the peak concentration would be 0.225 mM with a single 250 mg dose, assuming an average human adult blood volume of 5 L.

Systemic administration of acetazolamide might also be effective against the disseminated and fatal forms of *A. castellanii* infections, such as GAE, due to the permeability of the blood–brain barrier to acetazolamide.^50,51^ Anwar et al. utilized a similar approach for testing clinically used drugs to search for amoebicidal agents for treating manifestations in the central nervous system.^52^ Diazepam, phenobarbitone, and phenytoin had some amoebicidal and cysticidal effects,^52^ although the drugs were only tested for 24 h and excystation can take up to 36 h.^53^ The extended duration of our inhibitor assay allows the inhibitors to degrade over time, resulting in little inhibitory effect on cyst survival and thus representing the situation after ceasing antiamoebic treatment.

Ethoxzolamide is a commercially used pharmaceutical product presently in many countries, for example, in the United States. It is used in clinical work as an oral drug to treat glaucoma and duodenal ulcers. We selected the concentrations for ethoxzolamide based on maximum water solubility. Ethoxzolamide has better solubility in other solvents than water such as dimethyl sulfoxide. Still, water was selected for this experiment to produce conditions comparable to those of the other tested water-soluble compounds.

Fc14-584B is an experimental compound recently created as a β-CA inhibitor and a novel candidate to treat drug-resistant tuberculosis.^54^ The concentration of Fc14-584B was selected based on zebrafish survival tests, where a 300 μM concentration showed minimal adverse side effects on zebrafish larvae, and the LC50 dose was 498.1 μM.^54^

Brinzolamide is a clinically used eye drop with a concentration of 10 mg/mL (equivalent to 26.1 mM), and the maximum concentration in our experiment was half of that likewise, for the propamidine eye drop (0.1%). Both brinzolamide and propamidine were prone to crystal formation, producing technical challenges caused by crystal violet stains attached to the formed crystals and the amoebae, subsequently leading to a potential bias in the results. To address these effects, the wells were inspected through a light microscope (magnification 40×), and those wells containing crystals were excluded from the analysis. Previous inhibition assays have used Trypan blue and a hemocytometer to determine the cell count,^23,24^ which overcomes the problems caused by crystal formation. However, using a hemocytometer introduces the risk that the medium sample in the hemocytometer does not necessarily represent the density of cells in the whole assay. This is not a limitation of our method in which the entire well is analyzed. Ortega-Rivas et al. created a sulforhodamine B (SRB) staining colorimetric assay for drug screening in vitro.^55^ The challenge linked to SRB staining is that it only adheres to proteins in trophozoites, leading to the complete exclusion of cysts from the assay, thus limiting its ability to determine infections. Even though the superiority of different drug screening assays has not been experimentally verified, the non-nutrient *Escherichia coli* plate assay has been suggested to function better than the LDH release assay, trypan blue and fluorescent staining.^56^

The absorbance values between control curves (0) differ from each other in the testing of different inhibitors (range 0.5–1.9 in the inhibitor assay). Many factors can influence this. However, the most significant factor is the division schedule of the trophozoites. The state in the division process of trophozoite could not be determined in the beginning of the experiments; thus, they could have been in different stages in different experiments. In light of the time spent in one mitosis (8–24 h), our time points (24, 48, and 72 h) are not extensive enough to allow direct comparison of absolute absorption values between inhibitors.

In the excystation assay, we interpret the excystation capacity indirectly by measuring the absorbance of the crystal violet-stained trophozoites. As such, we are not able to state, for certain, from the decreased A590 readings whether the excystation capacity or the cell division capacities of the excysted trophozoites are suppressed.

Generally, interassay coefficient variation values under 15% are considered acceptable. Unfortunately, some of our results exceed that limit, but we speculate that it might be because of our small number of replicates, and perhaps a larger sample size would change the matter for the better.

Analysis of mRNA expression data suggests an active role of CAs in the physiology and metabolism of *A. castellanii*. Gene expression levels of most CAs were higher than the average expression levels of all genes. It is likely that by inhibiting these crucial proteins, vital biological processes would be disrupted and potentially induce cell death. This has been the case in previous studies by Aspatwar et al.,^57,58^ Pan et al.,^59^ Rahman et al.,^60^ Del Prete et al.^61^ and Abutaleb et al.^62,63^

Endosymbionts invade most of the isolated *A. castellanii* strains. The exact impact of CAI against AK with endosymbionts is unknown as our culture was axenic. *A. castellanii* provides a favorable environment for the endosymbiont; thus, inhibiting *A. castellanii* might also harm the endosymbiont.

Because we observe at least one *A. castellanii* CA among each of the major clades of endosymbiont CAs in the phylogenetic analysis, inhibitors that downregulate the enzymatic activity of those *A. castellanii* CAs may also affect the related CAs of endosymbionts. The common existence of various endosymbionts within *A. castellanii* organisms and our phylogenetic results may together indicate that the expression of multiple isoforms belonging to three different CA enzyme families may be due to the horizontal gene transfer from prokaryote microbes to the amoeba. In fact, *A. castellanii* may represent the first protozoan in which three CA families have been described in the same species.

Our results provide good evidence that CAIs are promising new drug candidates for treating AK and other invasive infections, such as GAE. We have provided an innovative new method to test the antiamoebic effects of different compounds in vitro, with the result of finding promising novel candidate drugs. Dorzolamide and acetazolamide were found to be most advantageous, with minimum effective concentrations of 100 nM and 100 μM, respectively. These drugs are especially attractive because they are already in clinical use for eye disease and glaucoma, beginning from 1995 and 1952, respectively, and are well-tolerated. In vivo trials are needed to test their capability against *A. castellanii* infections.

## Materials and Methods

### Culture Initiation and Maintenance

*Acanthamoeba castellanii* (ATCC 30,010; American Type Culture Collection; Manassas, VA, USA) arrived as frozen ampules which were first thawed for 2–3 min at +35 °C in a water bath. Then, the contents of the ampule were immediately transferred into T25 tissue culture flasks (Thermo Fisher Scientific, Waltham, MA, USA) with 5 mL of ATCC Medium 712, consisting of proteose peptone, yeast, glucose (PYG), and additives, as recommended by cell provider. Amoebae were incubated at +25 °C with constant temperature monitoring to ensure stable growth conditions. The axenic cell culture was maintained twice a week by extracting the old medium from culture flasks and replacing it with 5 mL of fresh medium. The purity of the culture was ensured at every maintenance step by inspecting the flask contents through a light microscope (magnification 40×). All procedures were executed aseptically to prevent contamination.

### Inhibitors Used

We tested six different inhibitors: five CAIs and propamidine (Brolene 0.1%, Sanofi, Paris, France), a medication already used to treat AK. The tested CAIs were acetazolamide (Diamox 100 mg/mL, Mercury Pharma, Croydon, United Kingdom), brinzolamide (Azopt 10 mg/mL, Novartis, Basel, Switzerland), dorzolamide (Sigma-Aldrich), ethoxzolamide (Sigma-Aldrich) and Fc14-584B.^54^ Propamidine (Brolene), acetazolamide (Diamox) and brinzolamide (Azopt) were commercial eye drops manufactured for clinical use. The purity of such drugs is rigorously controlled and monitored as part of the manufacturing process to ensure safety and effectiveness. The purity of such pharmaceutical compounds is not publicly disclosed by the manufacturer. The purity percentages of dorzolamide and ethoxzolamide informed by the manufacturer (Sigma-Aldrich) were ≥98% and ≥96.5, respectively, as determined by high performance liquid chromatography. Fc14-584B (4-methylpiperazin-1-ylcarbamodithioate) is >95% of purity. To confirm the purity, we determined the nuclear magnetic resonance (^1^H NMR, ^13^C NMR, ^77^Se NMR) spectra using a Bruker Advance III 400 MHz spectrometer in DMSO-*d*_6_, mass spectrometry using a Varian 1200L triple quadrupole system (Palo Alto, CA, USA) equipped with electrospray source (ESI) operating in both positive and negative ions, analytical thin-layer chromatography (TLC) carried out on Merck silica gel F-254 plates, and flash chromatography purifications performed on Merck silica gel 60 (230–400 mesh ASTM).

A dilution series was created for each inhibitor: either 10-fold (acetazolamide, brinzolamide, and dorzolamide) or 2-fold (propamidine and Fc14-584B). For ethoxzolamide, we used a combination of 2-fold (high concentrations) and 5-fold (low concentrations). The inhibitors in powder form (acetazolamide, dorzolamide, ethoxzolamide, and Fc14-584B) were first diluted in Milli-Q water to obtain stock solutions. Then the desired concentrations of inhibitors were produced by adding fresh PYG medium into the desired amount of stock solution to minimize the possible effect of water in the inhibitor testing. The ready-to-use eye drops (propamidine and brinzolamide) were used as stock solutions. The drug-free control was added with PYG medium to meet the same volume as that used in the inhibitor-containing wells.

### Inhibitor Assay

After approaching a steady state in the culture, the cells were gently detached with a cell scraper (Sarstedt Inc., Newton, MA, USA) from the bottom of the culture flask. The inhibition tests were performed in 96-well plates (Corning Inc., Corning, NY, USA) containing 1000 cells in 240 μL of cell-medium-inhibitor solution/well.

Time points for detecting the inhibition effect were 24, 48, and 72 h, with a separate plate for each time point. After the desired time point was reached, the medium-inhibitor solution was carefully pipetted into a new empty well plate. The new plate wells were refilled with fresh medium (addition of approximately 50 μL). For the cyst survival assay, new plates were incubated at +25 °C for 5 days to ensure the excystation and transformation into a metabolically active trophozoite, as excystation lasts for 12–36 h^16,53^ and one round of mitosis takes 8–24 h,^18^ summing to a maximum of 60 h to complete the excystation and first mitosis. After 5 days, the medium was removed, and the plates were handled like the original plates, as described below.

100 μL aliquot of methanol (Sigma-Aldrich) was added to each well to fix the cells on the well walls. The plates were incubated for 15 min at room temperature, after which the methanol was removed by reversing them and letting them dry with the lid open for at least 2 h. For staining, 100 μL of 0.1% crystal violet solution (Merck, Darmstadt, Germany) was added to each well which was then shaken with a horizontal shaker at 300 rpm for 20 min. Subsequently, crystal violet was washed away by submerging the plate into distilled water 10 times, with one water change after 5 times. Between each wash, the plates were reversed to remove the washing water. After washing, the plates were allowed to dry with the lid open for at least 2 h.

The crystal violet was diluted in 100 μL of 10% acetic acid (Sigma-Aldrich) in each well. The plates were shaken at 300 rpm for 15 min. All wells were inspected with light microscopy (magnification 40×) to ensure that only the cells bind to the crystal violet stain. The density of cells was determined with a VICTOR3 1420 Multilabel Counter (PerkinElmer, Waltham, MA, USA) by indirectly analyzing how the stain absorbs 590 nm wavelength light with Wallac 1420 Manager (PerkinElmer, Wallac Oy, 1997–2005, version 3.00). The more intense the stain, the larger the absorbance and, accordingly, the more cells in the well. The cell count was not determined at the end of the experiments.

Two separate experiments were made for each CA inhibitor with at least three adjacent wells for each concentration and time point, with a total of 6 wells for every concentration at minimum (range 6–15). The number of sample replicates were 11 for acetazolamide, 11 for brinzolamide, 15 for dorzolamide, 6 for ethoxzolamide, 7 for Fc14-584B and 6 for propamidine. Each plate also had empty wells filled with sterile water to maintain humidity and prevent evaporation from the test wells. In addition, every plate had control wells (indicated with 0) in which no inhibitor was present with the cells.

### Excystation Assay

Scattered cysts were exposed to inhibitors in the excystation assay. The culture was grown beyond the peak density to stimulate cyst formation. Similar concentrations of inhibitors, as described for the inhibitor assay, were applied in 96-well plates. Next, the cysts were collected, and 1000 cysts were added to each well in the assay. The plates were incubated for 72 h at +25 °C in order to provide time for the cysts to excystate and the excystated trophozoites to begin multiplication. Subsequently, the plates were fixed and stained, and in the process, all remaining cysts were washed away. The absorbance was analyzed as described above.

### Statistical Analysis

Absorbance data were analyzed, and figures were generated with GraphPad Prism (1992–2020 GraphPad Software, LLC, version 9.0.0). Due to the small sample size and differences in 24- and 48 h-time points not being immediately evident, the distribution of the absorbance data was determined at 72 h. The normality of the results was confirmed using the Shapiro–Wilk test and visual assessment of a QQ plot. Unpaired *t* tests were conducted between the control curve (0) and different concentrations at 72 h. Like the inhibitor assay, we evaluated the normality of the excystation assay and, as a result, used unpaired *t* tests between the control (0) and concentrations of the inhibitors. Interassay coefficients of variation were calculated using Microsoft Excel (version 2309, build 16.0.16827.20278. Microsoft Corporation).

### Expression Analysis

König et al. performed transcriptional analysis of *A. castellanii* pre- and postinfection with *Protochlamydia amoebophila* with mRNA sequencing.^36^ RPKMs (Reads Per Kilobase Million) of expression data (https://www.ncbi.nlm.nih.gov/geo/query/acc.cgi?acc=GSE93891) from uninfected samples were extracted for CA genes and transformed into TPM (Transcripts Per Million) units, with the formula presented in Zhao et al.,^64^ and plotted with GraphPad Prism (1992–2020 GraphPad Software, LLC, version 9.0.0).

### Phylogenetic Analysis of AK Endosymbiont CAs

Separate BLAST searches using each of the eight known *A. castellanii* CAs (3 α, 3 β, and 2 γ) were performed using the http://uniprot.org/ Web server (parameters: target database, UniProtKB; E-Threshold, 0.0001; Matrix, BLOSUM 64; Filter, low-complexity regions; taxa ids, 5207, 40,324, 780, 287, 83,552, 1773, 1765, 1764, 33,882, 445, 562, 813, 1,521,255, 673,862, 281,120, 41,276, 55,080, 222) to retrieve the top 1000 matching hits which occur in taxa matching known *A. castellanii* endosymbionts^31,37,38^ documented to co-occur in AK infections. Amino acid (AA) sequences and associated annotations were subsequently downloaded for all 2046 identified genes (α, 3; β, 1030; γ, 1013) using the http://uniprot.org/(65) representational state transfer (REST) application programming interface (API) using custom Python scripts. Sequences with lengths of less than 100 AA were removed. The retrieved β- and γ-CA endosymbiont sequences were subsequently taken for further separate phylogenetic analyses, as described in the following text.

Using the UCLUST clustering algorithm of the Usearch^66^ (version 11.0.667) sequence analysis tool, all duplicate gene sequences were removed and then the remainder clustered to centroids at 70% identity (parameters: “-centroids-id 0.70”, and all others as default), producing a final 95 β-CA and 46 γ-CA representative endosymbiont genes. To the β and γ endosymbiont CA gene sets were added the three β-CA genes and two γ-Ca genes from *A. castellanii*, respectively, for subsequent phylogenetic analyses. The β- and γ-CA gene sets were then each aligned (parameters: “-amino”, and all others as default) using MUSCLE^67^ (version 5.1). The alignments were then each filtered of low information content regions with GBlocks (vers. 0.91b)^68^ (parameters: “–*t* = *p* −*b*2 = 50 −*b*3 = 20 −*b*4 = 3 −*b*5 = *a* −*d* = *n*”, and all others as default).

Phylogenomic inference by maximum likelihood was performed, for both β- and γ-CA gene sets with the IQ-TREE software^44^ (version 1.5.5) with 100,000 bootstrap replicates for SH-aLRT and 100,000 bootstrap replicates (parameters: “-st AA-alrt 100,000 -bb 100,000 -nt AUTO”, and all others as default). The automatic IQ-TREE run of ModelFinder, for fast model selection for accurate phylogenetic estimates, determined the best AA substitution model for the β-CA set to be “LG + R5”; where “LG” is the Le and Gascuel amino acid exchange rate matrix^69^ and “R5” is the FreeRate model^70,71^ for rate heterogeneity across AA sites, with 5 categories. For the γ-CA set, the best model was determined to be “LG + G4”; where “G” is the discrete Gamma model^72^ for rate heterogeneity across AA sites, with 4 categories. The resulting consensus trees for both β- and γ-CA sets were then visualized with the ETE toolkit^45^ (version 3.1.2).

### Database Search of Endosymbiont CAs

Using the http://uniprot.org/ (The UniProt Consortium 2017) representational state transfer (REST) application programming interface (API), the UniProt database was queried for all genes annotated with “carbonic anhydrase” or “carbonate dehydratase” within 48 known *A. castellanii* endosymbiont,^31,37,38^ using custom Python scripts. AA sequences were subsequently downloaded for all 715 identified genes. For each organism or taxa, all duplicate gene sequences were removed and then the remainder clustered to centroids at 80% identity (parameters: “- centroids -id 0.80”, and all others as default), using the UCLUST clustering algorithm of the Usearch (Edgar 2010) (vers. 11.0.667) sequence analysis tool, producing a final 81 representative endosymbiont genes (Table 2).

## Abbreviations

AA: amino acid
AK: acanthamoeba keratitis
API: application programming interface
ATCC: American Type Culture Collection
CA: carbonic anhydrase
CAI: carbonic anhydrase inhibitor
GAE: granulomatous amoebic encephalitis
PYG: proteose peptone, yeast, and glucose solution
REST: representational state transfer
RPKM: reads per kilobase million
SD: standard deviation
SRB: sulforhodamine B
TPM: transcripts per million
